# Viral aetiology of influenza-like illnesses and severe acute respiratory illnesses in Morocco, September 2014 to December 2016

**DOI:** 10.7189/jogh.12.04062

**Published:** 2022-07-23

**Authors:** Abderrahman Bimouhen, Zakia Regragui, Fatima El Falaki, Hassan Ihazmade, Samira Benkerroum, Imad Cherkaoui, Ahmed Rguig, Hind Ezzine, Touria Benamar, Soumia Triki, Youssef Bakri, Hicham Oumzil

**Affiliations:** 1 Laboratory of Human Pathologies Biology, Faculty of Sciences, Mohammed V University in Rabat, Morocco; 2 Directorate of Epidemiology and Disease Control, Ministry of Health, Morocco;; 3 National Influenza Center, Virology department, National Institute of Hygiene, Ministry of Health, Morocco;; 4 WHO country office of Morocco, Rabat, Morocco; 5 Pedagogy and Research Unit of Microbiology, School of Medicine and Pharmacy, Mohammed V University in Rabat, Rabat, Morocco

## Abstract

**Background:**

There is a scarcity of information on the viral aetiology of influenza-like illness (ILI) and severe acute respiratory infection (SARI) among patients in Morocco.

**Methods:**

From September 2014 to December 2016, we prospectively enrolled inpatients and outpatients from all age groups meeting the World Health Organization (WHO) case definition for ILI and SARI from 59 sentinel sites. The specimens were tested using real-time monoplex reverse-transcription polymerase chain reaction method for detecting 16 relevant respiratory viruses.

**Results:**

At least one respiratory virus was detected in 1423 (70.8%) of 2009 specimens. Influenza viruses were the most common, detected in 612 (30.4%) of processed samples, followed by respiratory syncytial virus (RSV) in 359 (17.9%), human rhinovirus (HRV) in 263 (13.1%), adenovirus (HAdV) in 124 (6.2%), parainfluenza viruses (HPIV) in 107 (5.3%), coronaviruses (HCoV) in 94 (4.7%), human bocavirus (HBoV) in 92 (4.6%), and human metapneumovirus (HMPV) in 74 (3.7%). From 770 samples from children under 5 years old, RSV (288, 36.6%), influenza viruses (106, 13.8%), HRV (96, 12.5%) and HAdV (91, 11.8%) were most prevalent. Among 955 samples from adults, Influenza viruses (506, 53.0%), and HRV (167, 17.5%) were most often detected. Co-infections were found in 268 (18.8%) of 1423 positive specimens, and most (60.4%) were in children under 5 years of age. While influenza viruses, RSV, and HMPV had a defined period of circulation, the other viruses did not display clear seasonal patterns.

**Conclusions:**

We found that RSV was predominant among SARI cases in Morocco, particularly in children under 5 years of age. Our results are in line with reported data from other parts of the world, stating that RSV is the leading cause of lower respiratory tract infections in infants and young children.

Acute respiratory tract infections (ARIs) are a major public health problem that leads to higher morbidity and mortality worldwide, with the highest burden of disease in developing countries. Young children, the elderly, the chronically ill, and immunocompromised persons are at imminent risk [1]. ARIs are the leading cause of death among children under five years of age and are responsible for 19% of deaths and around 8% of all disabilities [2,3]. Practically, 13% of deaths reported in Moroccan children under five years old in 2012 were caused by ARIs [4].

The etiological agents for acute respiratory infections include bacteria, viruses, and fungi [5]. Viruses are responsible for around 80% of respiratory infections. They are responsible for upper respiratory infections (rhinitis, laryngotracheitis) and for potentially severe lower respiratory infections (bronchitis, bronchiolitis, and pneumonia) [6]. Several viruses are implicated in respiratory tract infections, including influenza viruses (Flu A/B), respiratory syncytial virus (RSV), human adenoviruses (HAdV), human coronaviruses (HCoV), human rhinovirus (HRV), parainfluenza viruses (PIVs), and human metapneumovirus (HMPV) [7].

While influenza is considered the most common cause of viral respiratory infections among older adults [8], RSV is recognized as the main viral pathogen in children [9]. RSV infections are responsible for most cases of severe symptoms such as bronchiolitis, asthma exacerbation and pneumonia, and lead to higher hospitalization rates [10]. Other non-influenza viruses may cause respiratory tract infections with similar symptoms and clinical features. Thus, identifying the aetiological agent is usually difficult without laboratory testing [11].

Although Morocco’s influenza surveillance system was established in 1996 [12], little data are available on the circulation of other respiratory viruses. To address this gap, virological data from the country’s existing influenza surveillance system was used to investigate the characteristics and circulation of respiratory viruses in patients with acute respiratory infections either admitted in hospitals or seen at out-patient clinics in Morocco from 2014 to 2016.

## METHODS

### Study design and case definition

A sentinel-based influenza surveillance system was established by health authorities in the country in 1996 [12].

The country’s respiratory surveillance network includes two concomitant components. While the outpatient influenza-like illness (ILI) surveillance system accepts patients attending health facilities with mild symptoms of fever ≥38°C and cough with an onset of >10 days [13], the severe acute respiratory infection (SARI) surveillance system takes hospitalized patients with SARI with fever ≥38°C and cough with an symptom onset within the same period [13].

SARI virological surveillance is conducted throughout the year in eight public general hospitals located in the country’s main regions. All patients who meet the WHO case definition of SARI are recruited and relevant epidemiological data are recorded by medical staff. The ILI virological surveillance system conducts specimen collection and data recording from the first five cases meeting the WHO’s case definition for ILI daily in eight out-patient public clinics and fifty private physicians located in nine main cities across the country.

The study period lasted from September 1, 2014, to December 31, 2016.

### Sample and data collection

Nasopharyngeal (NP) and oropharyngeal (OP) specimens were collected from all patients fulfilling ILI or SARI case definitions. Tubes of viral transportation medium (ViCUM^®^) containing specimens were conserved at 4°C at the health services and were sent to the National Influenza Center within two days. A standardized form with patient-specific information (medical history, clinical symptoms, and demographic and epidemiological data) was completed by physicians or health workers.

### Nucleic acid extraction

Total nucleic acids were extracted automatically from 400 μL of samples, using a High Pure Viral Nucleic Acid Kit and iPrep instrument, according to the manufacturer’s instructions (Lifetechnologies, Carlsbad, USA). After that, ribonuclease P (RNase P) was considered as the internal control during specimen extraction. 100μL of extracted nucleic acids were stored at -70°C until processing.

### Real-time reverse transcriptase PCR

Detection of 16 respiratory viruses was performed in 5μL volume using Invitrogen® Superscript III Platinum^®^ One-step Reverse transcription polymerase chain reaction (RT-PCR), amplification and reaction conditions were made with an ABI 7500 Fast Sequence Detection System^®^in accordance with the protocols developed by Centers for Disease Control and Prevention (CDC; Atlanta, GA) and provided as part of a material transfer agreement that ensures privacy and non-publication.

### Statistical analysis

To describe the temporal distribution of positive cases, we aggregated results obtained by real-time RT-PCR by calendar month and week. Demographic, clinical, and virological data for all enrolled patients were entered into a database created using Epi Info 7.1 (CDC; Atlanta, USA). Group comparisons were performed using χ^2^ for the dependency/relationship between variables. *P*-values <0.05 were considered statistically significant. Data analysis was conducted using the same software.

## RESULTS

### Demographic characteristics

From September 1, 2014, to December 31, 2016, NP and OP swabs were collected from 2009 patients meeting the WHO’s case definition for ILI and SARI from all age groups from 59 sentinel sites. 1187 (59%) specimens were collected from ILI and 822 (41%) from SARI sentinel sites. A slightly higher proportion of specimens belonged to female than to male patients (51.3% vs 48.7%). The patients’ mean age in years was 23.78 years (SD±23.8) and the median age was 16.00 years (IQR = 0.08-99.00).

A total of 740 patients (36.8%) were younger than 5 years, 842 (41.9%) were aged between 5-24 years, 273 (13.6%) were aged between 25-64 years, while 127 (6.4%) were older than 65 years (**Table 1**). More than half of the specimens were collected from two of the eight sentinel regions, Fes-Meknes (n = 753, 37.5%) and Rabat-Sale-Kenitra (n = 555, 27.6%). Although patients were enrolled for this study throughout the year, most specimens were collected during the fourth and first quarters of the season (October-April).

**Table 1:** Demographic characteristics of patients with influenza-like illness (ILI) and severe acute respiratory infections (SARI), Morocco, 2014-2016

	N = 1187	N = 822	N = 2009
	**ILI n (%)**	**SARI n (%)**	**Total n (%)**
**Sex**			
Male	515 (43.4)	463 (56.3)	978 (48.7)
Female	672 (56.6)	359 (43.7)	1031 (51.3)
**Age (years)**			
0–4	203 (17.1)	537 (65.3)	740 (36.8)
5–14	182 (15.4)	44 (5.3)	226 (11.2)
15–24	518 (43.6)	98 (12.0)	616 (30.7)
25–64	198 (16.7)	75 (9.1)	273 (13.6)
65+	74 (6.2)	53 (6.5)	127 (6.4)
Missing	12 (1.0)	15 (1.8)	27 (1.3)
**Regions (sentinel sites)**			
Beni Mellal-Khenifra	99 (8.3)	88 (10.7)	187 (9.3)
Fes-Meknes	458 (38.6)	295 (35.9)	753 (37.5)
Laayoune-Dakhla	2 (0.2)	2 (0.2)	4 (0.2)
Marrakech-Safi	60 (5.1)	5 (0.6)	65 (3.2)
Oriental (Oujda)	52 (4.3)	51 (6.2)	103 (5.1)
Rabat-Sale-Kenitra	284 (24)	271 (33.0)	555 (27.6)
Souss-Massa (Agadir)	99 (8.3)	99 (12.1)	198 (9.9)
Tanger-Tetouan	133 (11.2)	11 (1.3)	144 (7.2)

### Virus detection

A total of 1423 of 2009 specimens tested for both ILI and SARI were positive for at least one virus, resulting in a 70.8% detection rate. Among all positive specimens, a single infection occurred in 1155 (81.2%) patients while co-infections were detected in 268 (18.8%) patients. Dual, triple, and quadruple co-infections occurred in respectively 237 (16.7%), 28 (2.0%) and 3 (0.2%) patients.

Influenza was the most common virus, detected in 612 (30.5%) positive specimens, followed by RSV (n = 359, 17.9%), HRV (n = 263, 13.1%), HAdV (n = 124, 6.2%), PIVs (n = 107, 5.3%), HCoV (n = 94, 4.7%), human bocaviruses (HBoVs) (n = 92, 4.6%), and HMPV (n = 74, 3.7%) (**Table 2**). While 44.3% (*P* < 0.001) of influenza positives were detected in patients over 5 years of age with mild symptoms, positive RSV (42.4%; *P* < 0.001) and HAdVs (13.0%; *P* < 0.001) detections were mostly observed during SARI infection in children under 5 years with respectively of positive detections of these two viruses in this age category (**Table 3**).

**Table 2:** Frequencies of virus detections by age category in Morocco, September 2014 – December 2016

Virus	Total of detections N (%)*	
	**≤5 years**	**>5 years**
Flu A/B	106 (13.8)	506 (53.0)
RSV	282 (36.6)	77 (8.1)
HAdV	91 (11.8)	33 (3.5)
HRV	96 (12.5)	167 (17.5)
HMPV	36 (4.7)	38 (4.0)
HBoV	56 (7.3)	36 (3.8)
HPIV-1	20 (2.6)	10 (1.0)
HPIV-2	8 (1.0)	6 (0.6)
HPIV-3	29 (3.8)	25 (2.6)
HPIV-4	4 (0.5)	5 (0.5)
HCoV-229E	6 (0.8)	9 (0.9)
HCoV-NL63	20 (2.6)	18 (1.9)
HCoV-HKU1	5 (0.6)	14 (1.5)
HCoV-OC43	11 (1.4)	11 (1.2)

Flu A/B – influenza viruses, HCoV-229E – human coronavirus 229E, HCoV-NL63 – human coronavirus NL63, HCoV-HKU1 – human coronavirus HKU1, HCoV-OC43 – human coronavirus OC43, HAdV - human adenovirus, HMPV – human metapneumovirus, HPIV-1 – parainfluenza virus 1, HPIV-2 – parainfluenza virus 2, HPIV-3 – parainfluenza virus 3, HPIV-4 – parainfluenza virus 4, RSV – respiratory syncytial virus

*Percentage of viruses detected equals the number of positives from each group/total number of positives.

**Table 3:** Prevalence (n, %) of the viruses frequently related to respiratory infections by age category and origin of specimens, Morocco, September 2014 – December 2016

	TOTAL		ILI		SARI		*P-*value
	**<5 y, N = 740**	**>5 y, N = 1242**	**<5 y, N = 203**	**>5 y, N = 972**	**<5 y, N = 537**	**>5 y, N = 270**	
Positive specimen, n (%)							
Inf A/B	106 (14,3)	502 (40.4)	53 (26.1)	431 (44.3)	53 (9.8)	71 (26.3)	<0,001
RSV	282 (38.1)	73 (5.9)	54 (26.6)	63 (6.5)	228 (42.4)	10 (3.7)	<0,001
HAdV	91 (12.3)	33 (2.6)	21 (10.3)	25 (2.6)	70 (13.0)	8 (2.9)	<0,001
HRV	96 (12.9)	164 (13.2)	15 (7.3)	128 (13.2)	81 (15.1)	36 (13.3)	0,225

ILI – influenza-like illnesses, SARI – severe acute respiratory illness, Inf A/B – influenza A and B, RSV – respiratory syncytial virus, HAdV – human adenovirus, HRV – human rhinovirus

*Some variables from age have not been included in the analysis owing to incomplete data.

The number and rates of positive specimens stratified by virus and clinical symptom (**Table 4****)** showed that fever (55.2%; *P* = 0.013), rhinitis (16.5%; *P* < 0.001), headache (21.4%; *P* < 0.001) and sore throat (23.7%; *P* < 0.001) were more often reported in patients infected with influenza A/B compared to those infected with other viruses. Cough was the most reported clinical sign for all prevalent viruses, especially for RSV-positive cases (90.8%; *P* < 0.001).

**Table 4:** Number and rates of positive specimens by virus and clinical symptom, Morocco, September 2014 – December 2016

	N (%)			
**Symptoms**	**RSV (n = 359)**	**INF A/B (n = 612)**	**HRV (n = 263)**	**HAdV (n = 124)**
Cough	326 (90.8)	509 (83.2)	231 (87.8)	104 (83.8)
Fever >38	176 (49.0)	338 (55.2)	121 (46.0)	66 (53.2)
Rhinitis	20 (5.6)	101 (16.5)	28 (10.6)	10 (8.0)
Sore throat	52 (14.5)	145 (23.7)	39 (14.8)	14 (11.3)
Headache	25 (6.9)	131 (21.4)	31 (11.8)	9 (7.2)

### Seasonal distribution

**Figure 1:**
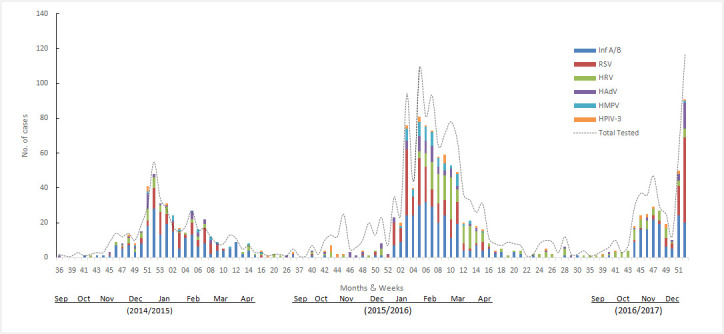
The incidence of the predominant respiratory viruses detected in Morocco, from September 2014 to December 2016. HAdV – human adenovirus, HMPV – human metapneumovirus, HPIV-3 – parainfluenza virus 3, RSV – respiratory syncytial virus, Inf A/B – influenza virus A and B, HRV – human rhinovirus

During the observation period, the study of seasonality showed that viral circulation extends throughout the year. However, there was a concomitant circulation of influenza and RSV viruses from November to April, with peaks during the months of December-March. HAdVs and HRV circulated throughout the year with peaks during the winter months. Other viruses often circulated during the cold season with sporadic cases throughout the year (**Figure 1**).

## DISCUSSION

In this study, we detected influenza and other respiratory viruses using RT-PCR in 2009 samples collected during the 2014/2016 influenza seasons through a sentinel-based influenza surveillance system. Among these samples, 1423 (70.8%) specimens were positive for at least one virus, which is consistent with the results from other studies with positivity rates between 63% to 75% [14-16].

According to WHO guidelines, the collection of patients’ specimens should be done rapidly after symptom onset, ideally within 7 days, and the specimens should reach the laboratory as soon as possible after collection [17]. It should be noted that viruses are generally detectable in throat swabs of most patients from the onset of symptoms until the end of the second week [17]. In our study, 72.0% (*P* = 0.005) of the positive samples were taken in the first week following the onset of the symptoms.

The findings also showed that Flu A/B (n = 612, 35.5%), RSV (n = 359, 20.8%), HRV (n = 263, 15.2%), and HAdV (n = 124, 7.2%) were the most common viruses detected. The representativeness of other viruses ranged from 5.3% to 0.5%. This is corroborated by one study [18], while others mentioned HRVs or HRSV as the most predominant viruses [14,16]. There are several explanations for these differences. First, the infection rates may be specific to geographic areas, size, type of sampling method, and study period. Second, detection methods and PCR primers may differ from one study to another, making it difficult to directly compare data. Other studies can clarify this question by evaluating the sensitivity and specificity of the different detection methods used [14,19].

Although influenza viruses were detected in all age groups, the proportion of positive cases for Flu A/B was comparatively higher in patients older than 5 years, especially in mild infections (88.7%; *P* < 0.001), which was consistent with similar studies conducted on adults that found influenza virus as the leading cause of ILIs among adults and that HRV was the second most common cause of ILIs in this category of age [15,18,20].

The most frequent viral pathogen in patients under five years old with SARI was RSV, followed by HAdV, HRV, and Flu A/B, as found in several studies [14,16,18,21-24].

The prevalence of RSV in children under 5 years of age with SARI was 42.4% (*P* < 0.001), which is high compared to data from other studies, which ranges from 18% to 34% [14,18,25]. This high prevalence of RSV infection among children can be attributed to the nosocomial spread of the virus within paediatric care units during the high circulating season of RSV. Despite the lack of data to support this hypothesis, some authors pointed to RSV as the major cause of annual nosocomial outbreaks, especially among the children admitted to paediatric units [26]. RSV infection is also recognized as a predominant problem in the elderly, especially among adults older than 65 years with chronic heart disease, asthma, lung disease, and immunocompromised patients. Despite the low number of patients recruited among adults over 65 years in this study, the prevalence of RSV infection was 7.1% in this age category, which is consistent with research suggesting a prevalence of RSV of approximately 5%-10% per year in the elderly [27].

Several studies [28-30] have revealed that the proportion of infections due to HAdVs was increasing. Indeed, in this present study the prevalence of HAdVs in SARIs among children under 5 years old was (13.0%; *P* < 0.001). This finding suggests that HAdVs was a significant pathogen of severe respiratory infections in hospitalized children.

Co-infections were observed in 18.8% (268/1423) of samples, which is coherent with rates reported in the literature ranging from 10% to almost 40% [31,32].

The samples co-infected with RSV and another virus constitute 45.9% (123/268) of the total mixtures. A 60.4% (162/268) co-infection rate was detected in children under 5 years old, which is consistent with several studies that suggest co-infections were significantly more widespread in paediatrics [31-34]. This is probably due to the children’s undeveloped immune system associated with the absence of respiratory virus infection history (primary infection with more than one virus at time) or RSV possibly facilitating the reinfection of the respiratory tract system by other viruses. Drew et al. [33] reported 85.7% of coinfections with RSV in children under 5 years old. Moreover, findings from other studies indicated that severe clinical cases were more common in patients with coinfections, particularly with RSV co-infections, which may increase the severity of the disease in children [34-36].

While influenza viruses, RSV, and HCoVs showed a seasonal peak during the winter season in temperate regions, HRV, HMPV, HBoV, and HAdV display no discernible seasonal patterns and can be considered all-year viruses [37].

In Morocco, the circulation of influenza viruses peaks in the period from October through April [12]. The detection rates for RSV and HMPV increased during the winter season and predominated along with influenza viruses. HRVs and HAdVs tend to circulate during the fall months, while HCoVs, PIVs and HBoV were circulating all over the year with no distinct seasonal patterns.

Our study has some limitations. First, specimen collection is limited to patients who fulfil the standard ILI/SARI symptoms according to World Health Organization ILI/SARI case definition, which may have excluded many viral infections and resulted in an underestimation of the detection rates of respiratory virus infections. Second, the small sample size did not allow a thorough investigation of the association between clinical and epidemiological characteristics and risk factors in relation to detected pathogens. Moreover, to establish the seasonality of the respiratory viruses circulating patterns, more time-series data analyses are required over 3 to 5 years periods.

## CONCLUSIONS

Our findings facilitate a better understanding of the characteristics and circulating patterns of respiratory viruses in patients with ILI or SARI in Morocco over the period of 2014-2016. While Influenza viruses are the most frequently detected respiratory viruses among outpatient adults, RSV remain the most important viral aetiological agent causing both mild and severe acute respiratory infections among children under 5 years old.

The co-circulation of several respiratory pathogens that cause infection with often similar symptoms requires greater vigilance and regular updating of the national epidemiological and virological surveillance system. Further research is needed to better understand respiratory viral epidemiology in our country, which may be useful for clinicians interested in the treatment and control of viral respiratory infections.

The recent COVID-19 pandemic showed the importance of whole-genome sequencing (WGS), hence the interest in developing the sequencing of emerging viruses. The combination of virological data issued from WGS and epidemiological data will provide precious information for public health decision-making to better manage potential epidemics and minimize socio-economic damage.
